# Nano-fenretinide demonstrates remarkable activity in acute promyeloid leukemia cells

**DOI:** 10.1038/s41598-024-64629-w

**Published:** 2024-06-14

**Authors:** Giovanna Farruggia, Lorenzo Anconelli, Lucrezia Galassi, Manuela Voltattorni, Martina Rossi, Pietro Lodeserto, Paolo Blasi, Isabella Orienti

**Affiliations:** 1 Department of Pharmacy and Biotechnology, University of Bologna, Via San Donato 19/2, 40127 Bologna, Italy; 2 Center for Applied Biomedical Research (CRBA), University of Bologna, 40126 Bologna, Italy; 3 National Institute of Biostructures and Biosystems, Via Delle Medaglie d’Oro 305, 00136 Rome, Italy; 4 Section of Endocrinology and Metabolic Diseases, Department of Systems Medicine, University of Rome Tor Vergata, 00133 Rome, Italy

**Keywords:** APL, 4-Hydroxyphenyl retinamide, *All-trans* retinoic acid, Antitumor activity, Mitochondrial membrane potential, Reactive oxygen species, HL60, HL60R, TK6, Cancer, Haematological cancer, Leukaemia, Acute myeloid leukaemia

## Abstract

Acute promyelocytic leukemia (APL) is characterized by rearrangements of the retinoic acid receptor, RARα, which makes *all-trans* retinoic acid (ATRA) highly effective in the treatment of this disease, inducing promyelocytes differentiation. Current therapy, based on ATRA in combination with arsenic trioxide, with or without chemotherapy, provides high rates of event-free survival and overall survival. However, a decline in the drug activity, due to increased ATRA metabolism and RARα mutations, is often observed over long-term treatments. Furthermore, dedifferentiation can occur providing relapse of the disease. In this study we evaluated fenretinide, a semisynthetic ATRA derivative, encapsulated in nanomicelles (nano-fenretinide) as an alternative treatment to ATRA in APL. Nano-fenretinide was prepared by fenretinide encapsulation in a self-assembling phospholipid mixture. Physico-chemical characterization was carried out by dinamic light scattering and spectrophotometry. The biological activity was evaluated by MTT assay, flow cytometry and confocal laser-scanning fluorescence microscopy. Nano-fenretinide induced apoptosis in acute promyelocytic leukemia cells (HL60) by an early increase of reactive oxygen species and a mitochondrial potential decrease. The fenretinide concentration that induced 90–100% decrease in cell viability was about 2.0 µM at 24 h, a concentration easily achievable in vivo when nano-fenretinide is administered by oral or intravenous route, as demonstrated in previous studies. Nano-fenretinide was effective, albeit at slightly higher concentrations, also in doxorubicin-resistant HL60 cells, while a comparison with TK6 lymphoblasts indicated a lack of toxicity on normal cells. The results indicate that nano-fenretinide can be considered an alternative therapy to ATRA in acute promyelocytic leukemia when decreased efficacy, resistance or recurrence of disease emerge after protracted treatments with ATRA.

## Introduction

Fenretinide (4-hydroxyphenyl retinamide, FEN) is a semisynthetic derivative of ATRA endowed with antitumor activity and a low toxicity profile. The main adverse effect is represented by decreased night vision reversible upon treatment suspension^1^. FEN antitumor activity, largely proved in a wide range of solid tumors and hematologic malignancies^2^ is based on multiple mechanisms triggering apoptosis in tumor cells by reactive oxygen species (ROS) increase, unbalancement of ceramides/dehydroceramides ratio and RARβ induction^3^.

Many studies reported FEN activity on non-APL, acute myeloid leukemia (AML) cells, without significant effect on normal counterparts^4–10^.

However, in spite of its antitumor activity and low toxicity profile, FEN has not entered in clinical use because its scant bioavailability prevents the achievement of suitable plasma concentrations to elicit a therapeutic response. The many attempts made over the years to increase FEN bioavailability produced only few formulations that entered in clinical trials but did not provide satisfying results either in solid tumors or in hematologic malignancies^2,3^.

A FEN nanoformulation (NF) was obtained by FEN entrapment in supramolecular aggregates, formed in aqueous medium by spontaneous self-assembling of suitable amphiphilic phospholipid-based mixtures. NF was studied in vitro and in vivo in a wide range of tumor models^11–16^. NF in vivo studies demonstrated a bioavailability and an antitumor activity much higher than free FEN, either by intravenous or oral administration^11–16^.

In this study we evaluated the activity of this nanoformulation in APL cell lines. AML is a malignant clonal disease where the presence of genetic alterations in myeloid cells causes inhibition of differentiation and arrest at varying stages of maturation. This makes AML characterized by several cell populations at various differentiation stages, expressing a variety of cell surface antigens and responding differently to therapy^17^. APL is a subtype of AML characterized by a chromosomic translocation that fuses the promyelocytic leukemia gene (PML) to the retinoic acid receptor alpha gene (RARα), resulting in PML-RARα oncogene^18,19^.

The fusion PML/RARα oncoprotein inhibits promyelocytes differentiation by binding to retinoic acid response elements (RAREs) and recruiting co-repressor proteins and histone deacetylases to form a complex that represses the expression of RARα target genes^20^. Treatment of APL with ATRA induces promyelocytes differentiation by linking RARα and inducing dissociation of the co-repressor complex^21^.

It has been observed that the use of ATRA in monotherapy provides complete remission in APL at high rate. However, over long-term treatment, resistance and decreased efficacy frequently occur due to induced metabolism, and relapse of disease appears within 3 to 6 months. In addition, fatal retinoic acid syndrome can occur in some cases^22^.

ATRA in combination with arsenic trioxide, with or without chemotherapy, results more effective than ATRA monotherapy. Indeed, ATRA with arsenic trioxide is currently the first-line treatment in APL, providing superior results of event-free survival and overall survival, and a significantly lower incidence of relapse than both ATRA monotherapy and ATRA in combination with chemotherapy^23^.

However, the use of ATRA at high doses and for prolonged time periods, as required in APL treatment, often provide a decline of the drug activity over time. This is due to self-induction of ATRA metabolism by the increased expression of cytochrome P450^24^ and RARα mutations triggered by prolonged body exposure to the drug^22^.

In addition, ATRA-induced differentiation can be reversed, and the cell reversion to the undifferentiated phenotype can reproduce the disease^25^. The differentiation syndrome (DS), a life-threatening complication, can occur in patients with APL undergoing induction therapy with ATRA or arsenic trioxide. DS affects about 20–25% of the patients and is mainly characterized by respiratory distress, cardiac involvement, hypotension, and/or acute renal failure. It is due to endothelial cell damage caused by maturing myeloid cells^26^. Another complication is the well-recognized neurotoxicity induced by arsenic trioxide^27–29^.

Despite these drawbacks, related to long-term treatments, the high efficacy of ATRA in APL has prompted a large amount of experimental work on synthetic retinoids for treatment of AML and other tumors.

Recent studies demonstrated that retinoids with high affinity for specific isoforms of RAR (RAR isoform-specific retinoids)^30,31^ or RARγ agonist retinoids^32^ are endowed with superior activity than ATRA in non-APL, AML treatment, due to the ability to induce differentiation in all the population of AML.

Many studies reported FEN activity on non-APL, AML cells, without significant effect on normal counterparts^5–11^. Only one paper reports the efficacy of FEN on an APL cell line in vitro^33^.

For these reasons we planned to firstly compare the activity of FEN with ATRA in the APL cell line HL60. Then we studied the effects of nanoencapsulated FEN and ATRA, to evaluate if the nanoformulation, an effective tool to obtain a high bioavailability after oral/intravenous administration, influenced the activity of the free drugs. Finally we evaluated nanoencapsulated FEN in APL cells lines resistant to doxorubicin to assess its suitability in chemotherapy-induced resistant APL, as an alternative to ATRA or as a tool for combination chemotherapy.

## Materials and methods

### Chemicals

FEN was purchased from Olon Spa (Milan, Italy), ATRA, soy L-α-phosphatidylcholine, glyceryl tributyrate, 2-hydroxypropyl beta cyclodextrin (Mw 1460) and KOH from Sigma-Aldrich (Merk Life Science S.r.l. Milan, Italy), ethanol absolute anhydrous was purchased from Carlo Erba Reagents (Milan, Italy). RPMI 1640 medium, dihydrodichlorofluorescein diacetate (H_2_DClFDA), Hoechst 33342, trisodium citrate, RNase, IGEPAL®CA-630, propidium iodide, glutamine, trypsine/EDTA solutions, were from Sigma-Aldrich Italia (Merk Life Science S.r.l. Milan, Italy). Foetal Bovine Serum (FBS) was from Euroclone (Milan, Italy).

### Preparation of FEN nanomicelles (NF) and all trans retinoic acid nanomicelles (NA)

NF were prepared according to a method previously reported^12,13^. Briefly, an amphiphilic semisolid phase was prepared by homogeneously mixing soy phosphatidylcholine (4 mmoles), glyceryl tributyrate (2 mmoles), 2-hydroxypropyl beta cyclodextrin (0.4 mmoles) and KOH 10 N (400 µL, 4 mmoles). FEN (1.2 mmoles) was dissolved in ethanol (300 µL) and KOH 10 N (120 µL, 1.2 mol) and was subsequently added to the semisolid phase. The resultant mixture was dispersed in PBS pH 7.4 to 50 mg/mL, filtered through 0.2 µm filters, and dialyzed for 72 h (dialysis membrane Mw cutoff 10 k Da, Fisher Scientific, Milan, Italy) against PBS pH 7.4. The dialyzed phase was lyophilized. The dry residue was reconstituted with water and filtered again through 0.2 µm filters to obtain 50 mg/mL NF, representing the final dispersion that was stored at − 22 °C until use. NA were prepared by mixing soy phosphatidylcholine (4 mmoles), glyceryl tributyrate (2 mmoles), 2-hydroxypropyl beta cyclodextrin (0.4 mmoles), KOH 10 N (400 µL, 4 mmoles) and adding ATRA (1.2 mmoles) previously dissolved in in ethanol (300 µL) and KOH 10 N (120 µL, 1.2 mol). The mixture was subsequently dispersed in PBS pH 7.4 to 50 mg/mL, filtered through 0.2 µm cellulose acetate filters (Fisher Scientific, Pittsburgh, PA, USA), and dialyzed for 72 h (dialysis membrane Mw cutoff 10 kDa, Fisher Scientific, Milan, Italy) against PBS pH 7.4. The dialyzed phase was lyophilized. The dry residue was reconstituted with water and filtered again through 0.2 µm filters to obtain the 50 mg/mL NA dispersion that was stored at − 22 °C until use.

### Characterization of the nanomicelles

The loading of FEN and ATRA in the nanomicelles was evaluated as previously described^12,13,16,17^. Briefly, the reconstituted nanomicelle dispersion (50 mg/mL) was diluted (1:3, v/v) with an ethanol:water (1:1, v/v) mixture and analyzed for drug content in comparison with the empty nanomicelles. The contents of FEN and ATRA were obtained by UV spectroscopy (Shimadzu UV-1601) at 360 nm and 295 nm respectively.

Particle size, polydispersity, and zeta potential were measured at 37 °C (Nicomp 380/ZLS, Particle Sizing Systems, Santa Barbara, CA, USA) on the reconstituted nanomicelle suspensions (50 mg/mL) after dilution with PBS to 0.05 mg/mL. A minimum of 12 measurements per sample were made. Results were the combination of 3, 10-min runs for a total accumulation correlation function time of 30 min. The results were volume-weighted.

The nanomicelle stability to drug leakage was measured by the release of FEN or ATRA from NF and NA, respectively, as previously described^12,13,16,17^. Briefly, the reconstituted nanomicelles suspensions (50 mg/mL) were diluted to 0.05 mg/mL with pH 7.4 PBS containing FBS (10% v/v) and 1 mL of the diluted suspension was placed in a releasing chamber separated from a receiving compartment by a dialysis membrane (Mw cutoff 5 kDa, Fisher Scientific, Milan, Italy). The receiving compartment was filled with 10 mL of pH 7.4 PBS containing FBS (10% v/v). Leakage from the nanomicelles was determined by spectrophotometric determination of FEN or ATRA in the receiving phase at their maximum absorption wavelengths. The system was maintained at 37 °C and sink conditions were monitored throughout the experiment.

### Cell lines

Human promyelocytic leukemia cell line HL60 was purchased from American Type Culture Collection (ATCC, Manassas, VA), its MDR subline resistant to doxorubicin (HL60R) was kindly provided by M.L. Dupuis (Istituto Superiore di Sanità, Roma, Italy) and TK6 limphoblasts were kindly provided by M. Lenzi (Department of Pharmacy and Biotechnology University of Bologna, Italy). For the present study the cells were grown in RPMI 1640 supplied with 10% FBS and 2 mM glutamine at 37 °C in a 5% CO_2_ humidified atmosphere. They were maintained between 1 × 10^5^ and 1 × 10^6^ cell/mL.

### MTT assay

The tetrazolium salt 3-(4,5-dimethylthiazol-2-yl)-2,5-diphenyl tetrazolium bromide (MTT) assay was used to detect cell proliferation and viability. Briefly, this method is based on reduction of MTT to the insoluble formazan salt by cellular dehydrogenase. The amount of formazan produced is usually considered as a good indicator of the number of viable cells in the sample^34,35^. However, the different metabolic and energetic status of the cells^36–38^ can influence their ability to reduce MTT to formazan thus affecting the results. Similar considerations can be applied to assays using other reducible substrates, such as Alamar Blue. Other methods to analyze cell proliferation relay on monitoring cell number over time in the presence of viability dyes. These methods are time-consuming and require sophisticated instrumentations. Therefore, MTT assay remains a good indicator of cell cytotoxicity^34,35^, and, being one of the most used, the results obtained can be easily compared with other reported in the literature.

To perform the MTT assay, the cells were seeded at 1 × 10^5^ cell/mL in 96 multiwell plates, and, after 1 h, they were treated with NF, NA, or the free drugs for 24, 48 or 72 h. Afterwards 10 µL of a 5 mg/mL MTT solution were added to each well to a final concentration of 0.5 mg/mL. After 4 h at 37 °C in the dark, 100 µL of a solution containing 10% (w/v) sodium dodecylsulfate (SDS) and HCl 10 mM were added to each well to dissolve the insoluble purple formazan crystals and left overnight on a shaker. The absorbance of each well was read on a TECAN plate reader (Männedorf, Switzerland) at 570 nm.

The cells were treated with NF or NA at concentrations expressed as weight of nanoparticles/mL, ranging from 0.15 µg/mL to 150 µg/mL. These amounts corresponded to FEN or ATRA concentrations ranging from 0.03 to 30 µM. Therefore, treatment with the nanoformulations were referred, thruought the text, as μM concentrations of FEN and ATRA administered as NF and NA, respectively. The free drugs were tested, as a comparison, in the same concentration range (0.03 µM to 30 µM) as the nanoencapsulated drugs. Starting solutions of the free drugs in ethanol (10 mM) were used after dilution with cell medium.

### Cell cycle analysis

HL60 cells were seeded at 1 × 10^5^ cell/mL in 6 multiwell plates and treated with NF or free FEN for 4, 24, 48 and 72 h or with NA or free ATRA for 72 h. After treatment the cells were counted and stained according to the protocol reported by Erba et al.^39^ with minor adjustments. Briefly, 1 × 10^6^ cells were centrifuged at 250×*g* 10 min and the pellet was suspended in trisodium citrate 0.1% w/v, RNase 10 μg/mL, IGEPAL®CA-630 0.01% v/v, and 50 μg/mL propidium iodide (PI). After 30 min of incubation at 37 °C in the dark, cells were analyzed through a BRYTE HS Flow Cytometer (Bio-Rad, Hercules, CA, USA) equipped with a Xe/Hg lamp tuned at 480 nm. PI fluorescence was collected with an emission band centered at 600 nm, on a linear scale. DNA distribution in the cell cycle was analyzed by the Modfit software version 5.2 (Verity, San Jose, CA, USA).

### Reactive oxygen species determination

The fluorescent assay with H_2_DClFDA is widely used to evaluate intracellular ROS production. H_2_DClFDA entrapped within the cells is hydrolized by cellular esterase and converted to fluorescent dichlorofluorescein (DClF) by the action of ROS. Even if DClF is mainly produced by H_2_O_2_, it is now well known that it can be oxydated by other ROS, as HO**·** and ROO**·**^40,41^.

The cells (1 × 10^6^) were treated with NF, or free FEN at concentrations corresponding to 0.25 µM and 0.5 µM FEN, for 4 or 24 h. ROS production was evaluated by staining the cells with H_2_DClFDA. Briefly, 0.5 × 10^6^ cells were centrifuged at 250×*g* 10 min and the pellet was suspended in serum free medium containing H_2_DClFDA 1 µM. The cells were incubated in the dark for 30 min at 37 °C, washed and resuspended in PI 5 µg/mL in PBS to selectively stain dead cells. Flow cytometry data were acquired using S3 BioRad Flow cytometer or a Brite HS Flow cytometer (BioRad). The yielded fluorescences were acquired and plotted on a logarithmic scale, and DClF production was gated on viable cells (PI negative). ROS production was evaluated as the mean channel of DClF fluorescence and reported as fold increment of fluorescence with respect to the mean channel of control. Typical plots of negative control, positive control and treated cells are reported in SI_1 (Fig. and Table).

### Mitochondrial membrane potential

The cells (1 × 10^6^) were treated with NF or free FEN at concentrations corresponding to 0.25 µM and 0.5 µM FEN, for 4, 24, 48 or 72 h. The mitochondrial membrane potential was evaluated by incubating the cells with the metachromatic cationic dye JC1 1.5 μM for 15 min at 37 °C, washing and analyzing by S3 BioRad Flow Cytometer. JC1 accumulation is dependent on the mitocondrial potential: if a sufficient concentration is achieved, JC1 forms so called J-aggregates which fluoresce in red, while monomers fluoresce in green.

Mitochondrial membrane depolarization was indicated by a decrease in the red (590 ± 10 nm) to green (525 ± 25 nm) fluorescence intensity ratio. Red and green fluorescence were collected on a logarithmic scale. Typical cytograms of control and treated cells are reported in SI_2 (Fig. and Table) and the percentage of cells in the region R7 (polarized mitochondria) and in the region R9 (depolarized mitochondria) were evaluated.

### Confocal laser-scanning fluorescence microscopy

Confocal laser scanning microscopy (CLSM) is a useful tool for imaging living or fixed cells containing fluorophores. High-resolution images are generated by photons emission from the excitated fluorophores. To obtain images with CLSM, the cells (1 × 10^5^ cell/mL) were treated with NF or free FEN at concentrations corresponding to 8 µM FEN for 4 h, or with NF, free FEN, NA, free ATRA at concentrations corresponding to 1 µM FEN or ATRA respectively for 24 h. Nuclei were stained with 1 µg/mL Hoechst 33342 for 30 min at 37 °C in the dark. The cells were subsequently washed with PBS three times, fixed with 2% formaldehyde for 10 min at room temperature, washed repeatedly with 0.1 M glycine/PBS. The cell were sedimented on a glass slide by citospin at 200×*g* 10 min. Specimens were embedded in Mowiol and analyzed using a Nikon C1s confocal laser-scanning microscope, equipped with a Nikon PlanApo 40, 1.4-NA oil immersion lens. FEN was excited at 405 nm with an argon laser and emission was recorded 650 nm. Hoechst fluorescence was excited at 405 nm and the emission was recorded at 440 nm. The images were analyzed by Image J Software (version 1.53a, U. S. National Institutes of Health, Bethesda, MD, USA).

### Statistics

All experiments were repeated at least three times on three independent samples. Statistical analysis was performed by one-way analysis of variance (ANOVA) followed by Dunnett’s multiple comparison test. Differences of *p* < 0.05 were considered significant. GraphPad Prism Software (version 9, GraphPad Software, San Diego, CA, USA) was employed.

## Results

### Characterization of the nanomicelles

**Figure 1:**
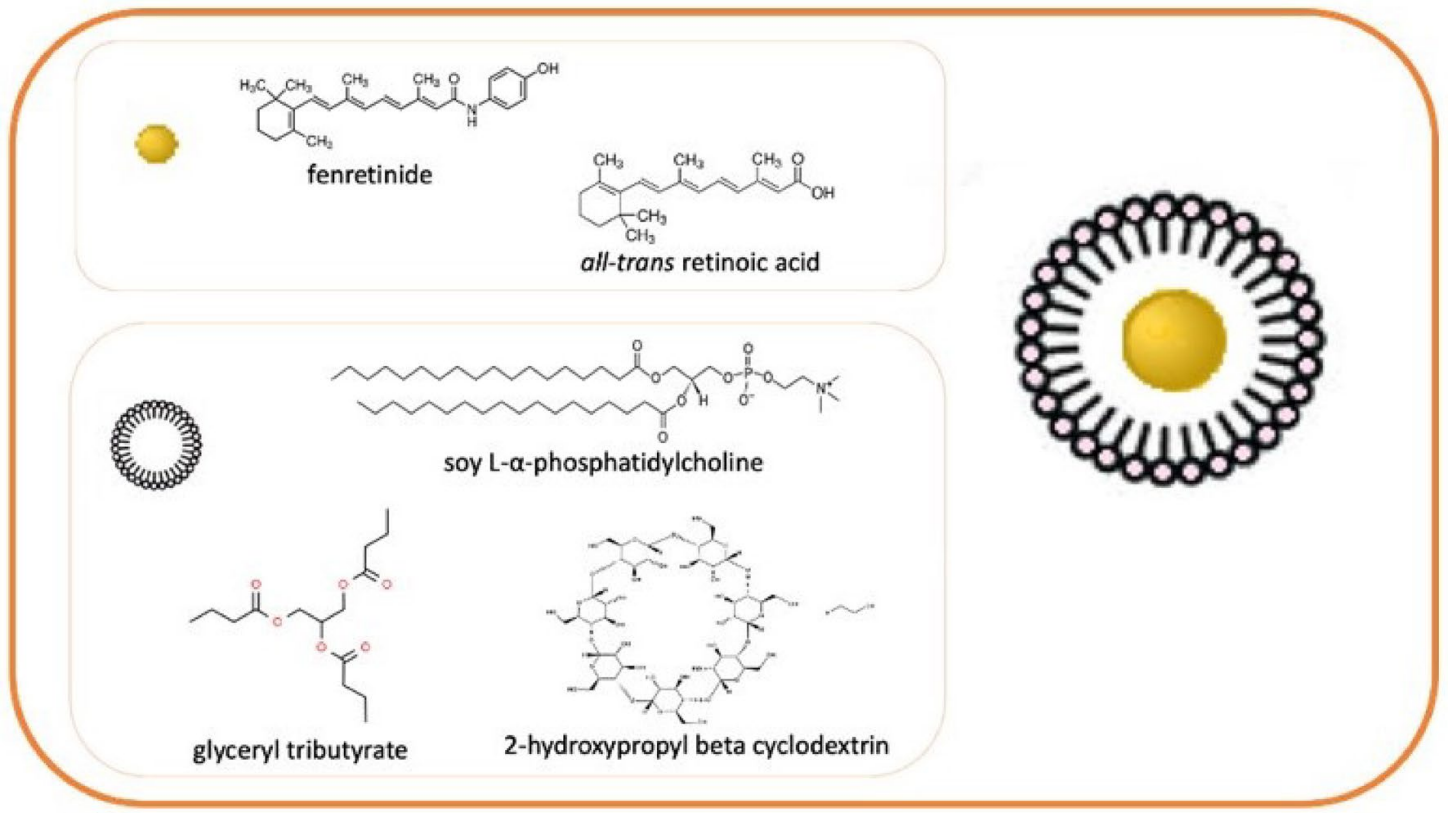
Schematic representation of nanomicelles entrapping FEN or ATRA. The nanomicelles were obtained by an amphiphilic mixture based on phospholipids, glyceryl tributyrate and 2-hydroxypropyl beta cyclodextrin.

The nanomicelles were obtained spontaneously by dispersing in PBS a mixture of phospholipids, glyceryl tributyrate, 2-hydroxypropyl beta cyclodextrin, FEN or ATRA in defined molar ratios. The amphiphilic character of the mixture triggers a supramolecular assembly in an aqueous environment, with formation of nanomicelles that entrap FEN or ATRA^11–16^. In Fig. 1, a schematic cartoon is reported.

Drug loading, mean hydrodinamic diameter, polydispersity, zeta potential and drug leakage were measured on suspensions of NF and NA at 0.05 mg/mL in PBS containing FBS (10% v/v) to simulate the concentrations of the nanomicelles in the medium used for the cytotoxicity experiments. The results showed that the nanomicelles were suitable for tumor accumulation thanks to the EPR (enhanced permeability and retention) effect^42–46^. In fact their size is less than 300 nm and polydispersity index less than 0.2, corresponding to a homogenous size distribution. The nanomicelles carried a negative surface charge, as indicated by the zeta potential values (Table 1).

**Table 1:** Physicochemical characteristics of NF and NA in PBS at 0.05 mg/mL. Data are reported as mean ± standard deviation.

Formulation	Loading (% w/w)	Mean Hydrodynamic diameter (nm)	Polydispersity Index	Zeta Potential (mV)	Drug Leakage at 24 h (%)
NF	8.03 ± 0.91 [FEN]	209.7 ± 2.41	0.18 ± 0.01	− 25.5 ± 1.57	16.3 ± 2.79
NA	7.69 ± 0.34 [ATRA]	94.9 ± 0.20	0.16 ± 0.003	− 41.3 ± 0.59	4.70 ± 1.05

The zeta potential of NA was more negative than NF due to the presence of ATRA carboxyl residues. Both NF and NA were stable to drug leakage, the release being less than 20% of the drug loading after 24 h. This is a necessary feature to convey the drug to the tumor site with high efficiency and avoid unwanted release of the drug during circulation.

### Treatment of HL60 with FEN and ATRA

#### Cell viability

Treatment of HL60 cells was performed with NF and free FEN in comparison with NA and free ATRA since leukemic promyelocytic cells are known to be responsive to ATRA. NF and NA were used at concentrations ranging from 0.15 µg/mL to 40 µg/mL corresponding to FEN and ATRA concentrations ranging from 0.03 µM to 8 µM.

**Figure 2:**
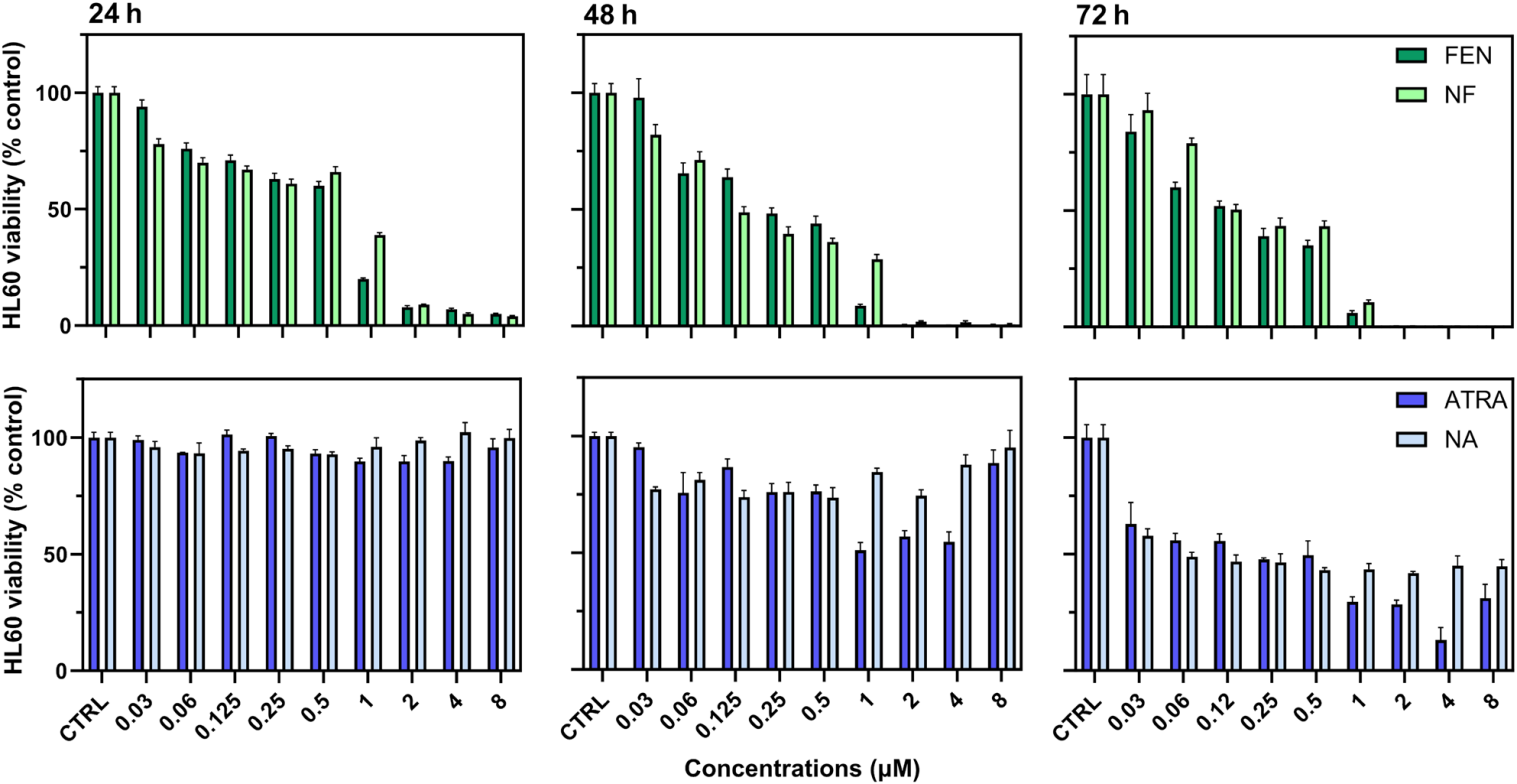
Relative viability of HL60 cells treated with increasing concentrations of free FEN (dark green bar), NF (light green bar), free ATRA (dark blue bar) or NA (light blue bar) for 24, 48 and 72 h. Viability was extimated by MTT assay and expressed as percentage versus control (100%) (mean ± SD, n = 6).

Treatment with NF provided a progressive decrease in cell viability. After 24 h a slight decrease was observed up to 1 µM, reaching about 40% the control viability. Beyond 1 µM a sharper decrease was obtained with the viability reduced to less than 10% the control (Fig. 2). Similar results were found 48 h after treatment. A stronger effect was obtained 72 h after treatment since 1 µM reduced viability to about 3%.

A similar trend was obtained with free FEN at lower concentrations than NF (Fig. 2). This effect increased over time particularly with NF where the drug release from the nanomicelles is expected to be delayed by the nanomicelles disassembling within the cells.

To compare the effect of FEN with the standard therapy, HL60 cells were treated with ATRA and NA at the same concentrations used with FEN and NF. ATRA and NA decreased cell viability only at 72 h, providing a maximum viability decrease of about 50% at 0.5 µM NA, without any further viability decrease at higher concentrations, as shown in Fig. 2. Free ATRA provided slightly higher activity than NA at concentrations higher than 1 µM. However, the differences were not significant (Fig. 2). Surprisingly, both ATRA and NA were less active at the highest concentrations used (4 µM and 8 µM) than at lower concentrations, probably due to lower drug availability caused by oversaturation. These results further support a much higher activity of FEN than ATRA. Based on the results obtained, the IC50 were evaluated for FEN and NF and are reported in Table 2.

**Table 2:** IC_50_ concentrations (μM) of FEN and NF on HL60 cells at 24, 48 and 72 h.

Time (h)	Treatment	IC50 (μM)	Confidence Interval
24	FEN	0.4538	0.3036–0.6685
	NF	0.8242	0.4656–1.4160
48	FEN	0.2705	0.1890–0.3848
	NF	0.1824	0.1285–0.2686
72	FEN	0.1633	0.1152–0.2338
	NF	0.2082	0.1449–0.3028

#### Cell cycle

To evaluate the influence of treatments on the cell cycle we treated the cells with FEN and NF at concentrations slightly higher than the IC_50_ values obtained at 72 h, and with ATRA and NA at the highest concentrations used (4 µM and 8 µM).

**Figure 3:**
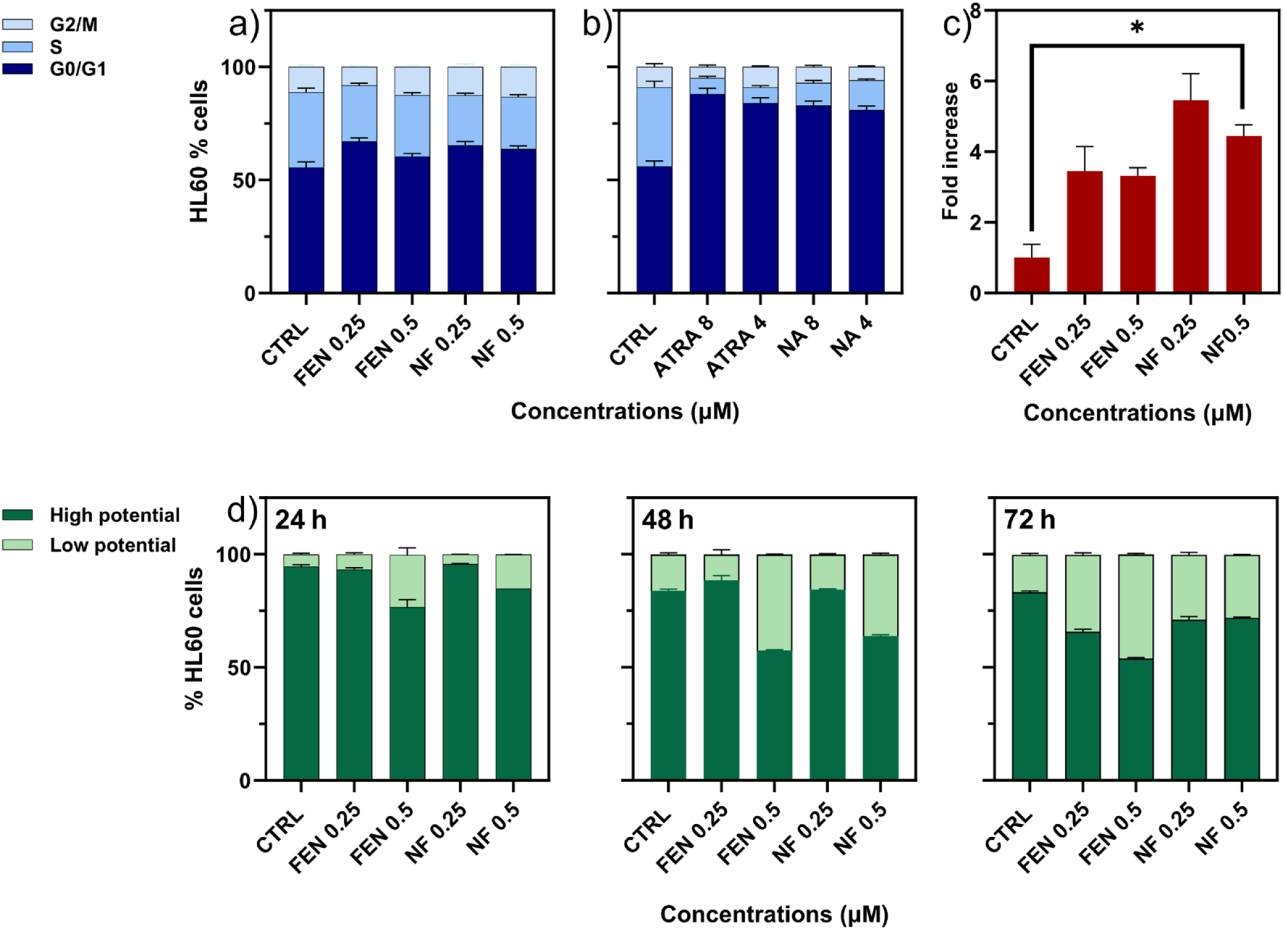
Cytofluorimetric data. Effect of (**a**) FEN and NF, (**b**) ATRA and NA on cell cycle evaluated by cytofluorimetric assay. The percentage of HL60 cells in the different phases are reported for different treatment concentrations after 72 h. Dark blue, dim blue and light blue bars indicate the cell percentages in the G0/G1, S, G2/M phase respectively. (**c**) ROS production in HL60 treated with different concentrations of free FEN or NF for 4 h. Increment in ROS production was evaluated by DCF assay and expressed as fold increase with respect to control taken as 1 (mean ± SD, n = 6) (*p < 0.05). (**d**) Mitochondrial potential of HL60 cells treated with free FEN or NF for 24 h, 48 h, 72 h. Dark green and light green bars indicate cell percentage with high potential and low potential respectively (mean ± SD, n = 6) (**d**).

The results reported in Fig. 3a,b indicated that at 72 h there is no effect on cell cycle for FEN and NF, while a high increase in G0/G1 phase was obtained with ATRA and NA. This is in accordance with the differentiation activity of ATRA on HL60 cells.

#### ROS production

ROS production was evaluated in HL60 cells after 4 h and 24 h of treatment with NF and FEN. As shown in Fig. 3c, after 4 h of tratments both FEN and NF increased ROS levels. This increase was relevant at 4 h while after 24 h the ROS levels decreased to values similar to the untreated cells.

#### Mitochondrial potential

Mitochondrial potential provides informations on the cell ability to produce ATP. A mitochondrial disfunction gives a decrease in mitochondrial potential and this alteration is known to induce cell death. As reported in Fig. 3d, treatment with FEN and NF provided a gradual increase over time of a cell population characterized by low potential indicating a progressive damage of the mitochondrial cells function. NF increased the low potential cell population less effectively than FEN at each time in accordance with the requirement of FEN to be released from the nanomicelles to become effective.

#### Morphologic evaluation of apoptosis

**Figure 4:**
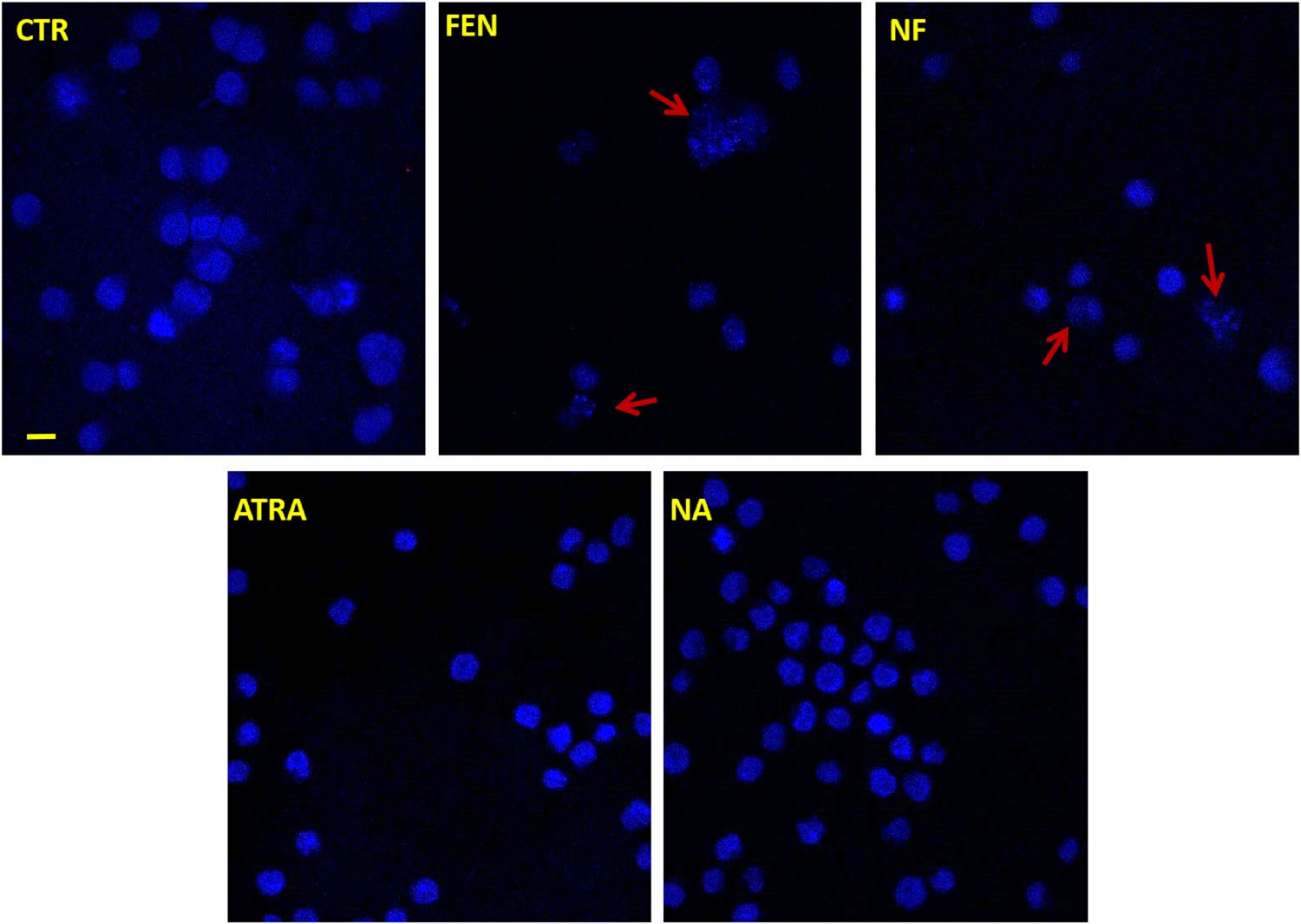
Confocal microscopy to evaluate the nuclear morphology of control HL60 cells (CTR) and treated cells for 24 h with free FEN, NF, ATRA and NA. Apoptotic nuclei are highlighted by arrows. Images were recorded at 40 × magnification, bar = 10 µm.

Cells treated for 24 h with NF and FEN at 1 µM have been imaged by confocal microscopy. The images showed in Fig. 4 revealed that both NF and FEN provided an evident nuclear fragmentation indicative of apoptosis. Treatment with ATRA and NA on the contrary did not show any apoptosis sign being the nuclei homogeneous and without fragments.

### Treatment of HL60R with FEN

**Figure 5:**
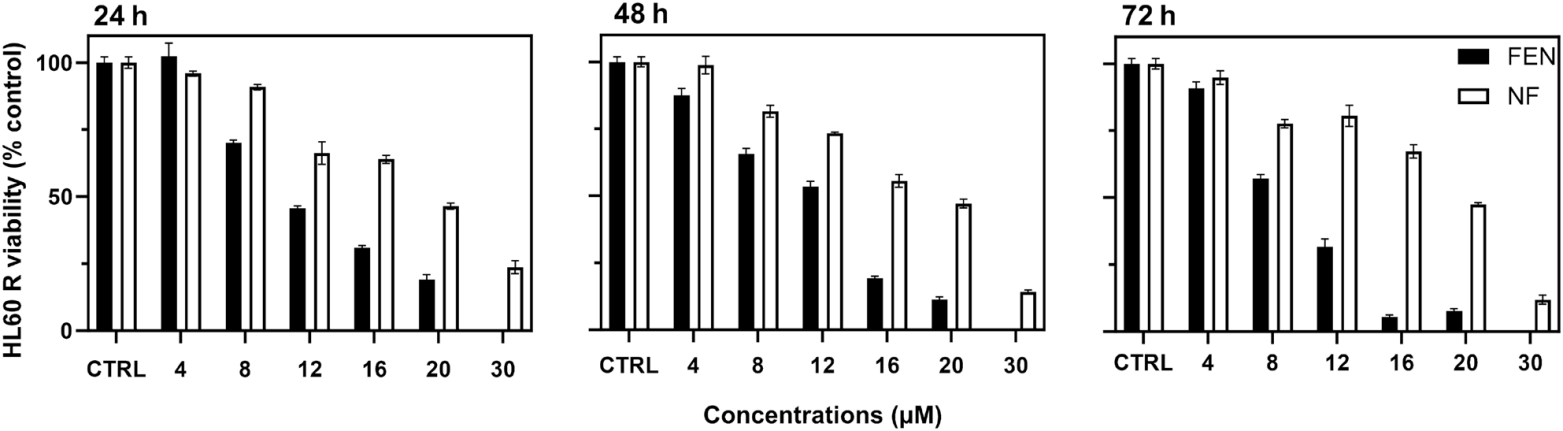
Relative viability of HL60 R cells treated with increasing concentrations of free FEN and NF for 24 h, 48 h and 72 h. Viability was evaluated by MTT assay and expressed as percentage versus control (100%) (mean ± SD, n = 6).

Treatments with NF and FEN were less effective in HL60R cells than in HL60. While in HL60 a complete decrease in viability after 72 h of treatments was achieved with 1 µM FEN and NF (Fig. 2), in HL60R comparable decreases in cell viability were obtained at 16 µM FEN and 30 µM NF as shown in Fig. 5. The decreased activity in HL60R may be correlated with decreased penetration and retention of FEN and NF into the cells due to the main features of multidrug resistance, such as membrane rigidification and overexpression of drug efflux P-gp, that have been demonstrated in resistant promyelocytic leukemia cells^47–50^:

Additionally, the lower activity of NF than FEN is in agreement with the larger nanoparticles size, limiting their penetration in the rigidified membranes, with respect to the much smaller size of the free drug molecule.

### Treatment of TK6 with FEN

TK6 lymphoblasts were used as a model for normal blood cells to evaluate the possible cytotoxic effects of NF and FEN on the healthy cells of blood^51^.

NF and FEN did not significantly decrease TK6 cells viability at concentrations up to 1 µM, which is highly active in HL60 (Fig. SI_3). These data indicated that FEN is less cytotoxic to normal than tumor cells and the drug encapsulation in nanomicelles further decrease the drug cytotoxicity to normal cells.

### Penetration of FEN in cells

The tumor cells HL60, HL60 R and the normal TK6 lymphoblasts were exposed to NF or FEN at 8 µM for 4 h and analyzed by confocal laser-scanning fluorescence microscopy.

**Figure 6:**
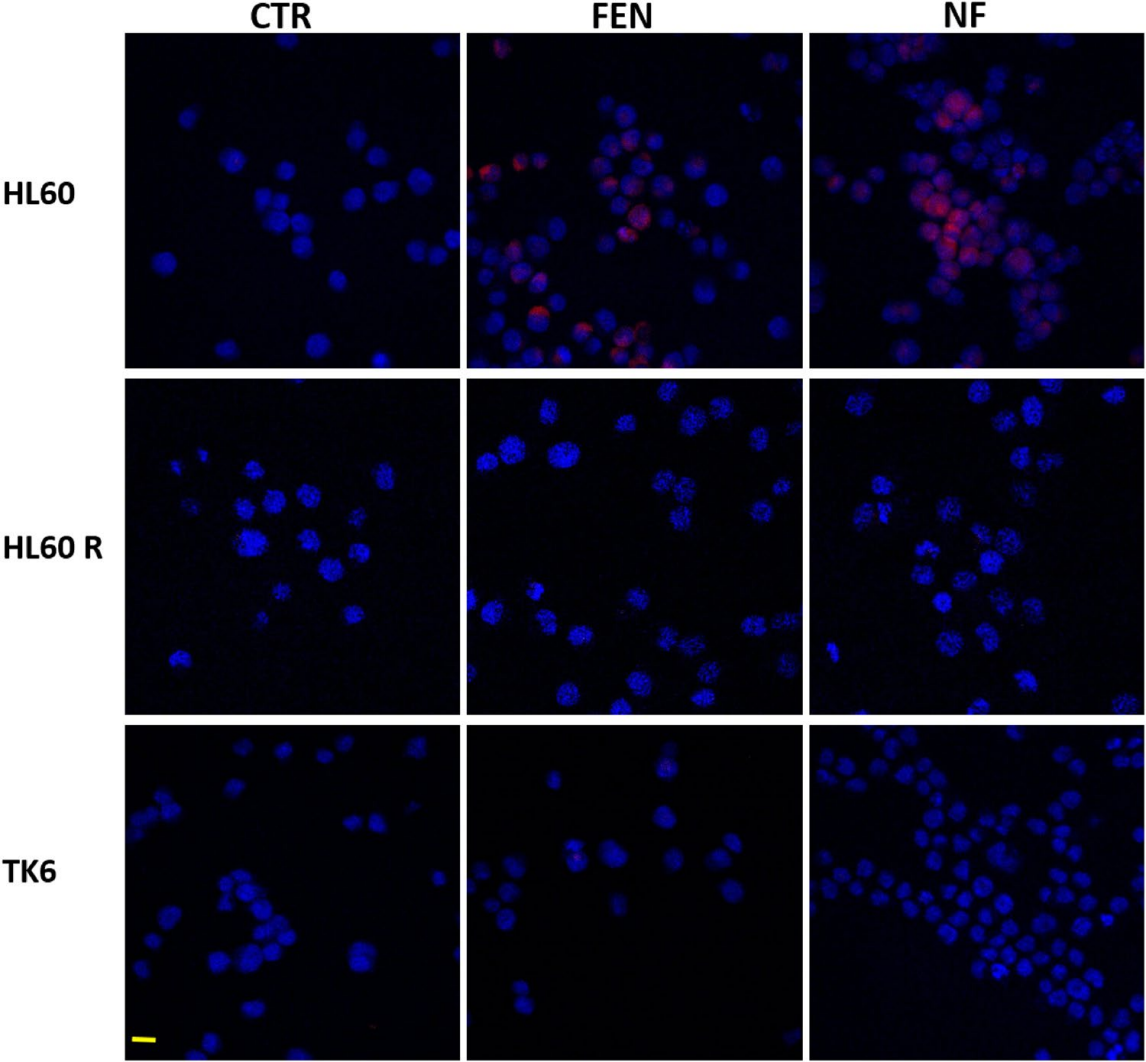
Confocal microscopy of HL60, HL60R, TK6 cells after 4 h treatment with free FEN (FEN) or NF at 8 µM at 40 × magnification, bar = 10 µm.

As evidenced in Fig. 6, red fluorescence, revealing FEN presence within the cells, was observed in HL60 treated by both FEN and NF, while no fluorescence was detected in HL60R and TK6 lymphoblasts. This indicated that FEN can penetrate HL60 cells as a free drug or loaded in nanomicelle and penetration is a rapid process being evident after a short-term treatment.

In HL60R, on the contrary, no red fluorescence was observed in the cells after treatment. In this case, the structural and functional changes induced by resistance, in particular the membrane rigidification, can account for inhibition of FEN penetration either as nanomicelles or as a free drug. The images of TK6 lymphoblasts did not show any FEN penetration accounting for a more stable and less permeable membrane in normal cells than their tumor counterparts^42^.

## Discussion

The activity of FEN in AML cells has been widely described in the literature^4–10^. It has been correlated to NF-κB inhibition and induction of NR4A1 translocation from nuclei to mitochondria with block of Bcl-2 anti-apoptotic proteins. The high tolerability of this drug has been demonstrated in preclinical and clinical studies on patients with solid tumors and hematologic malignancies^3^. However, in spite of its antitumor activity and low toxicity profile, FEN has not entered in clinical use because its scant bioavailability prevents the achievement of suitable plasma concentrations to elicit a therapeutic response. The many attempts made over the years to increase FEN bioavailability produced only few formulations that entered in clinical trials but did not provide satisfying results neither in solid tumors nor in hematologic malignancies^2,3^.

A nanotechnology-based formulation, NF, was obtained by FEN entrapment in self-assembling supramolecular aggregates. This system is spontaneously formed by dispersing in water an amphiphilic mixture of phosphatidylcholine, glyceryl tributyrate and 2-hydroxypropyl beta cyclodextrin. NF has been studied in vitro and in vivo in a wide range of tumor models^11–16^. The results demonstrated high bioavailability and antitumor activity either by intravenous and oral administration^11–16^.

At the best of our knowledge, no studies have been performed in hematologic malignancies. Therefore, in the present work we evaluated the in vitro activity of NF in acute promyelocytic leukemia.

We found that NF was very active in HL60 with IC_50_ of about 0.2 µM at 72 h. This concentration was much lower than the active concentrations previously found in cell lines of solid tumors where the IC_50_ values were in the range 1–5 µM in neuroblastoma, 1–10 µM in glioblastoma and lung cancer, 1–20 µM in colon cancer and melanoma^13,14^.

Previously published in vivo studies^11–13^ indicated that FEN concentrations achievable in plasma and tumors by oral or intravenous administration of NF are in the same range needed for in vitro activity. In particular, FEN plasma concentration of about 18 μM was obtained by a single NF oral administration corresponding to 200 mg/Kg FEN^12^, while FEN plasma concentrations of about 10 or 12 μM were obtained by single or repeated NF intravenous administrations (30 mg/Kg of FEN), respectively^12^. This indicates that a therapeutic activity in APL can be reasonably expected by single or repeated oral or intravenous administrations of NF.

The molecular mechanisms of FEN antitumor activity have been extensively evaluated in different cells types. The results indicated the drug ability to interfere with many metabolic routes and signaling pathways^3^ leading to ROS increase, ceramide/dihydroceramide dis-regulation and apoptosis.

In HL60 we found that the cytotoxic activity of NF was associated with the main mechanisms of FEN antitumor activity leading to apoptosis. Indeed we detected ROS increase, mitochondrial damage and signs of apoptosis, observed as nucleus fragmentation by confocal microscopy. No changes in cell cycle were found by treatment with NF.

ATRA was used as a comparison, since in APL a rearrangements of the retinoic acid receptor, RARα, makes ATRA highly effective and, in combination with chemotherapy or arsenic trioxide, it is currently used as a model of precision medicine^18,25,26^. Treatment of HL60 cells with ATRA, both as a free drug or encapsulated in the same type of nanomicelles used for FEN, did not increase ROS, or induce signs of apoptosis. It significantly increased the cell accumulation in the G0/G1 phase indicating differentiation.

Differentiation is the mechanism by which ATRA and ATRA-ATO combination provide excellent response rates in acute promyelocytic leukemia. However it is known that differentiatiated cells can revert, on a long term, to the undifferentiated phenotype^25^ triggering a relapse of disease. Moreover the differentiation syndrome can occur by long treatments with ATRA. It is a life-threatening condition due to endothelial damage linked to promyelocyte differentiation^26^.

Therefore, based on the results from this study, NF can be considered a better option than ATRA in the treatment of promyelocytic leukemia, due to its ability to induce apoptosis, instead of differentiation, and being characterized by antitumor activity at very low concentrations.

NF demonstrated activity also in doxorubicin resistant promyelocytic leukemia cells, but at higher concentrations than in sensitive cells. Indeed, the viability of HL60R cells was decreased to 10% by treatment with 30 µM NF at 72 h, while the same decrease was obtained in HL60 at 1 µM. The lower activity in resistant cells can be attributed to hindered penetration of FEN and NF, with respect to sensitive cells, as showed by confocal microscopy images. Indeed, the red fluorescence, indicative of FEN penetration, was evident in HL60 after a short period treatment and completely absent in HL60R.

The decreased penetration was due to cell changes related to multidrug resistance, a survival program activated by cells to counteract the cytotoxic activity of chemotherapic agents. The main functional changes in multidrug resistant cells are the overexpression of drug efflux P-gp and other ABC proteins, as well as membrane rigidification affecting plasma membrane permeability^47–50^.

Again, the NF concentrations active in HL60R cells corresponded to FEN levels easily achievable in vivo, as demonstrated by NF administration in mouse models of solid tumors^11–16^.

The cytotoxic activity in doxorubicin resistant promyelocytic leukemia cells suggests that NF can be useful to eradicate the minimal residual disease, characterized by the persistence of residual resistant cells, after chemotherapy, unresponsive to the majority of conventional treatments.

A comparison of NF with free FEN outlines the influence of nanoencapsulation in drug transport through cell membranes. The similar activity of NF and FEN in sensitive cells and the higher activity of the free drug in resistant cells is in accordance with membrane rigidification in resistant cells, restraining intracellular penetration to the large nanomicelles more than to the free molecule.

In normal lymphoblasts, nanoencapsulation decreased the effect of FEN which, at the highest concentrations, slightly reduced cell viability thus indicating that nanoencapsulation can mitigate the cytotoxic effects of the free drug to normal blood cells.

The role of nanoencapsulation in improving on-target activity of antitumor drugs has been widely demonstrated in solid tumors where leaking blood capillaries and inefficient lymphatic drainage promote accumulation and retention of intravenously injected drug-loaded nanoparticles^52–55^. The resulting high drug concentration, generated by nanoparticle accumulation in tumors, accounts for improved activity than free drugs.

Another important feature of nanoencapsulation is the improvement of bioavailability of ‘critical drugs’ such as FEN, whose very high hydrophobic character prevents its solubilization in plasma and body fluids, thus precluding in vivo use by conventional formulations.

In ‘liquid tumors’ such as leukemias, nanotechnology-based formulations have been barely studied. However they can be regarded as useful tools to target leukemic stem cells aggregates which frequently form in bone marrow niches and can cause relapse of the disease. The formation of stem cells aggregates is favoured by survival of tumor cells to therapy caused by scarce penetration of free drugs in bone marrow^56,57^. Accumulation of drug-loaded nanoparticles in bone marrow is a well-known event associated with nanoparticles injection for treatment of solid tumors. It is favored by nanoparticles extravasation through the physiological discontinuities of the bone marrow capillaries.

Thus, increased drug concentration and protracted drug release, generated by nanoparticles accumulation in bone marrow, can be expected to provide enhanced efficacy towards leukemic stem cell aggregates with overall improved therapeutic efficacy than free drugs.

Treatment with NF can therefore be regarded with interest in APL as it allows to exploit FEN accumulation in bone marrow as well as making FEN bioavailable at therapeutic levels for this disease.

## Conclusion

NF, a nanotechnology-based formulation of FEN, can be considered as a consistent alternative therapy in APL when decreased efficacy or resistance emerge after protracted treatments with *all-trans* retinoic acid. Also, the antitumor activity based on apoptosis, makes NF a useful tool in case of disease recurrence, due to myelocytes dedifferentiation towards a tumorigenic state, after suspension of *all-trans* retinoic acid treatment.

The concentrations levels of FEN suitable for reducing HL60 viability to 90-100% are easily achievable *in vivo* as indicated by previous pharmacokinetics and biodistribution studies in solid tumor models, where NF administration provided FEN plasma concentrations higher than the concentrations active *in vitro* in both HL60 and HL60R cell lines.

### Supplementary Information

Supplementary Information.

## Data Availability

All data are available on request to the corresponding authors.
