# Supplementary material for: Nano-fenretinide demonstrates remarkable activity in acute promyeloid leukemia cells
Source: Sci Rep. 2024 Jun 14;14:13737. doi: 10.1038/s41598-024-64629-w (PMC11178801; doi:10.1038/s41598-024-64629-w)
Supplement: Supplementary file 1 — Supplementary Information. [file 41598_2024_64629_MOESM1_ESM.docx]

**Supplementary material**

**Nano-Fenretinide Demonstrates Remarkable Activity in Acute Promyeloid Leukemia Cells.**

Giovanna Farruggia ^1,2,3^, Lorenzo Anconelli^1^, Lucrezia Galassi^1,2^, Manuela Voltattorni^1^, Martina Rossi^1,2^ , Pietro Lodeserto^4^, Paolo Blasi ^1,2*^ and Isabella Orienti ^1,^*

^1^Department of Pharmacy and Biotechnology, University of Bologna, Via San Donato 19/2, 40127 Bologna, Italy.

^2^Center for Applied Biomedical Research (CRBA), University of Bologna, 40126 Bologna, Italy.

^3^ National Institute of Biostructures and Biosystems, Via delle Medaglie d’Oro 305, 00136 Rome, Italy.

^4^ Section of Endocrinology and Metabolic Diseases, Department of Systems Medicine, University of Rome Tor Vergata, 00133 Rome, Italy.

***** Correspondence: [isabella.orienti@unibo.it](mailto:isabella.orienti@unibo.it); [p.blasi@unibo.it](mailto:p.blasi@unibo.it)

**SI_1. Flow cytometric assay of ROS production**


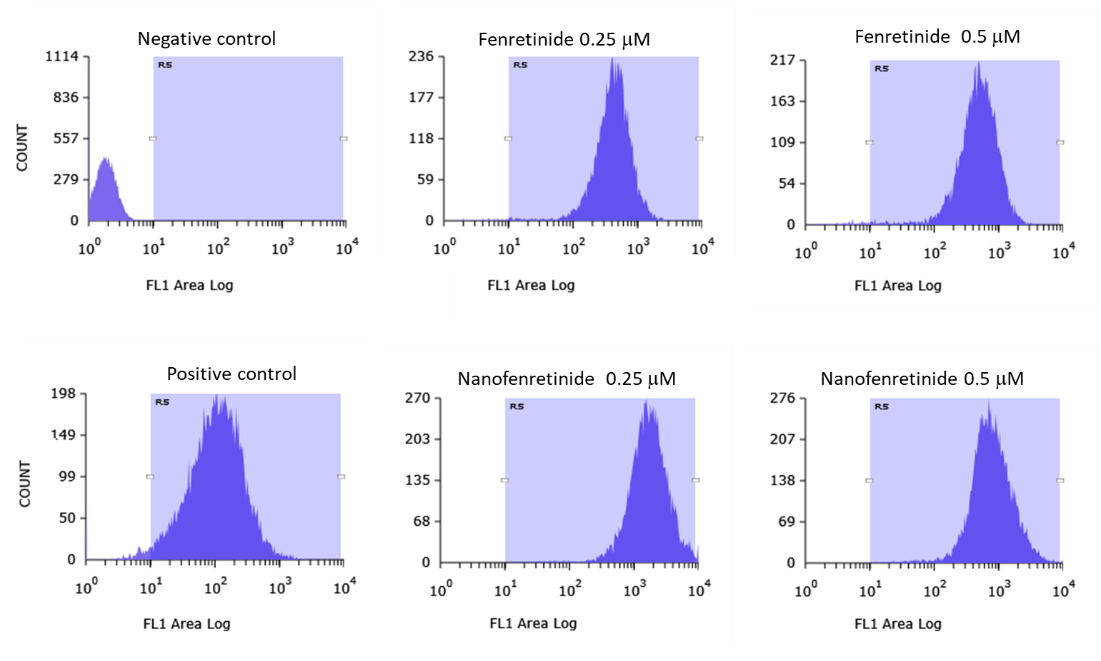


Figure SI_1. Flow cytometric evaluation of ROS production by DClF staining in a typical experiment: the cells were analyzed evaluating the mean fluorescence of DClF (FL1) entrapped in viable cells, as reported in Material and Methods section. The negative unstained cells were used to set the region 5 (R5), where the signals from the labeled cells fall. Data are reported in table S1

Table SI_1. Percentage of cells in Region 5 and mean channel of the DClF fluorescence distribution in Region 5 for the experiment in Fig. SI_1.

|  | **Negative Control** | **Positive Control** | **Fenretinide0.25 µM** | **Fenretinide**  **0.5 µM** | **Nanofenretinide 0.25 µM** | **Nanofenretinide 0.5 µM** |
| --- | --- | --- | --- | --- | --- | --- |
| % in R5 | 1.11 | 97.70 | 99.14 | 98.86 | 99.47 | 99.88 |
| Mean channel | 52.96 | 160.71 | 497.90 | 597.28 | 2080.41 | 1023.74 |

**SI_2. Flow cytometric assay of mitochondrial potential**


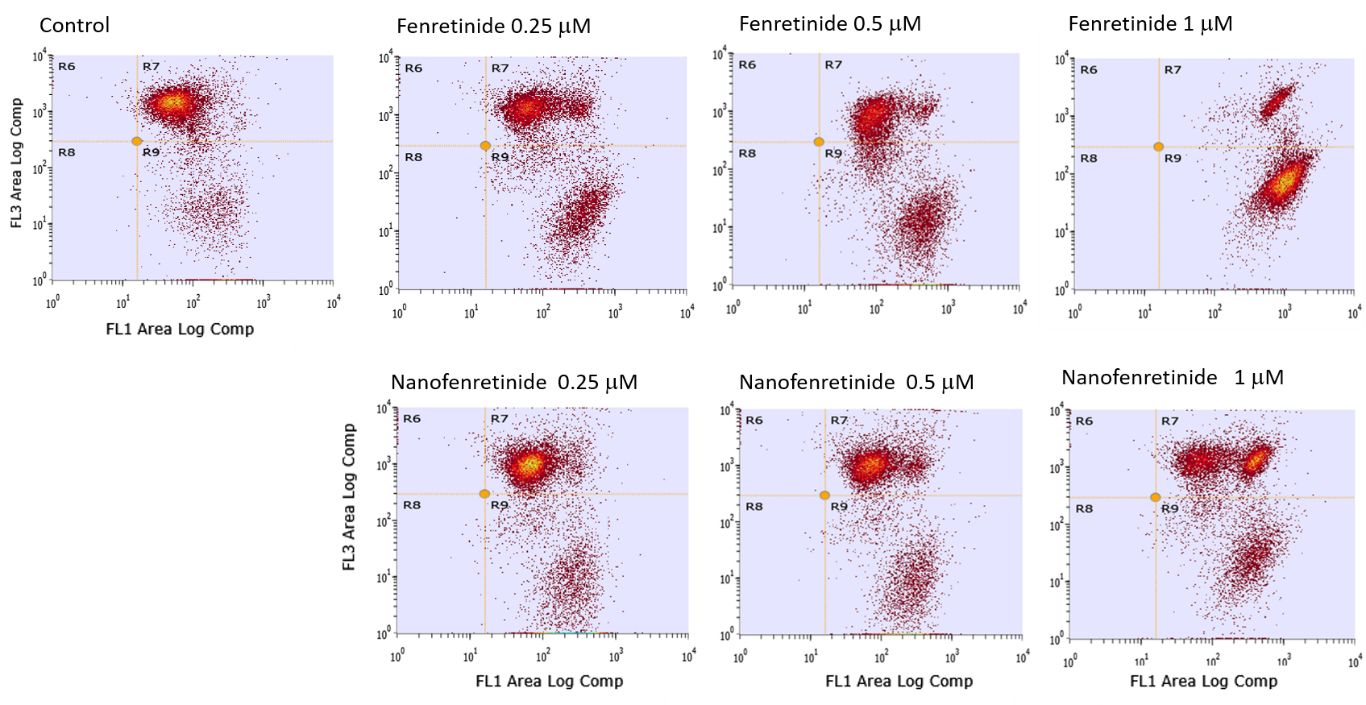


Figure SI_2. Flow cytometric evaluation of the mitochondrial potential by JC1 staining in a typical experiment. JC1 is a metachromatic probe that accumulates in the matrix as a function of mitochondrial potential. If the mitochondrion is active, the elevated dye concentration causes the formation of aggregates that fluoresce red (FL3), while the monomer fluoresces green (FL1). In the cytograms the percentage of cells with the different fluorescence can be evaluated in the regions R7 (high red fluorescence) and R9 (lower red fluorescence/higher green fluorescence). The results are summarized in table S2.

Table SI_2. Percentage of cells in region 7 and region 9 in the experiment in Fig. SI_2.

|  | **Control** | **Fenretinide 0.25 µM** | **Fenretinide 0.5 µM** | **Fenretinide1 µM** | **Nanofenretinide 0.25 µM** | **Nanofenretinide 0.5 µM** | **Nanofenretinide 0.5 µM** |
| --- | --- | --- | --- | --- | --- | --- | --- |
| % of cells in R7 | 81.87 | 63.20 | 50.97 | 17.07 | 67.71 | 68.63 | 69.10 |
| % of cells in R9 | 17.52 | 36.16 | 48.82 | 82.93 | 31.65 | 30.62 | 30.44 |

**SI_3. TK6 relative viability after treatment with FEN and NF**


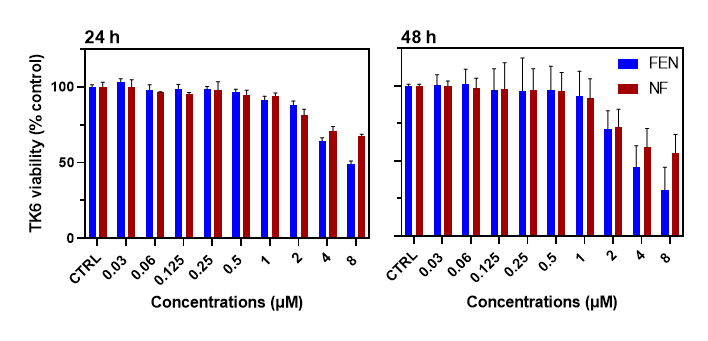


Figure SI_3. Relative viability of TK6 cells treated with increasing concentrations of free FEN and NF for 24 h and 48 h. Viability was evaluated by MTT assay and expressed as percentage versus control (100%) (mean ± SD, n = 6).
